# The Physician’s Visiting List for 1895

**Published:** 1894-12

**Authors:** 


THE PHYSICIAN'S VISITING LIST for 1895. P. Blakiston, Son & Co., Philadelphia, Pa. Price, for 25 patients per day or week, $1.00.

The forty-fourth annual edition of this visiting list is to hand. It contains a Calendar, the Metric or Decimal Systems, Posological Table, Dose Table, List of New Remedies, Poisons and Their Antidotes, Incompatibles, Examination of Urine, Diagnosis of Simple Diseases of the Eye, Table of Eruptive Fevers, etc., etc.

This little volume is truly a vade-mecum for the practitioner, as it contains in the smallest possible compass more useful and practical information than we have ever seen in any visiting list. G. B.

